# Temporal trends in non-occupational sedentary behaviours from Australian Time Use Surveys 1992, 1997 and 2006

**DOI:** 10.1186/1479-5868-9-76

**Published:** 2012-06-19

**Authors:** Josephine Y Chau, Dafna Merom, Anne Grunseit, Chris Rissel, Adrian E Bauman, Hidde P van der Ploeg

**Affiliations:** 1 Prevention Research Collaboration, Sydney School of Public Health, University of Sydney, Sydney, Australia; 2 School of Biomedical and Health Sciences, University of Western Sydney, Sydney, Australia

## Abstract

**Background:**

Current epidemiological data highlight the potential detrimental associations between sedentary behaviours and health outcomes, yet little is known about temporal trends in adult sedentary time. This study used time use data to examine population trends in sedentary behaviours in non-occupational domains and more specifically during leisure time.

**Methods:**

We conducted secondary analysis of population representative data from the Australian Time Use Surveys 1992, 1997 and 2006 involving respondents aged 20 years and over with completed time use diaries for two days. Weighted samples for each survey year were: n = 5851 (1992), n = 6419 (1997) and n = 5505 (2006). We recoded all primary activities by domain (sleep, occupational, transport, leisure, household, education) and intensity (sedentary, light, moderate). Adjusted multiple linear regressions tested for differences in time spent in non-occupational sedentary behaviours in 1992 and 1997 with 2006 as the reference year.

**Results:**

Total non-occupational sedentary time was slightly lower in 1997 than in 2006 (mean = 894 min/2d and 906 min/2d, respectively; B = −11.2; 95%CI: -21.5, -0.9). Compared with 2006, less time was spent in 1997 in sedentary transport (B-6.7; 95%CI: -10.4, -3.0) and sedentary education (B = −6.3; 95%CI: -10.5, -2.2) while household and leisure sedentary time remained stable. Time engaged in different types of leisure-time sedentary activities changed between 1997 and 2006: leisure-time computer use increased (B = −26.7; 95%CI: -29.5, -23.8), while other leisure-time sedentary behaviours (e.g., reading, listening to music, hobbies and crafts) showed small concurrent reductions. In 1992, leisure screen time was lower than in 2006: TV-viewing (B = −24.2; 95%CI: -31.2, -17.2), computer use (B = −35.3; 95%CI: -37.7, -32.8). In 2006, 90 % of leisure time was spent sedentary, of which 53 % was screen time.

**Conclusions:**

Non-occupational sedentary time has increased slightly from 1997 to 2006 in the Australian adult population. This seems to be the result of small increases in sedentary transport and education time while sedentary household and leisure time were stable over this time period. However, almost all leisure time is spent sedentary and the composition of sedentary leisure time changed between 1992 and 2006 towards a larger proportion being screen-based activities. This could be an important observation for public health, as most of the evidence on the detrimental effects of sedentary behaviour is around watching television and health.

## Background

Sedentary behaviours are a ubiquitous part of modern life. These are activities involving low energy expenditure (1–1.5 metabolic equivalents)[1] such as sitting at work, watching TV and driving a car, and are distinct from a lack of moderate-to-vigorous physical activity.[2] Time spent being sedentary may be associated with increased risk of chronic illness and mortality independent of physical activity [3-5].

Population surveillance indicates that the prevalence of sitting and sedentary behaviour is high. The International Prevalence Study of physical activity, which surveyed self-reported sitting times in 20 countries, found that adults report sitting for five to six hours per day on average.[6] Other population studies, which objectively assessed sedentary time with accelerometers, found that adults are sedentary for approximately 57 % of their waking time.[7,8] However, current prevalence data on sitting and sedentary behaviours are limited to single data collection periods with inadequate contextual detail about sedentary behaviour and so little is known about changes over time in adult sitting and sedentary behaviour in defined populations.

Time use surveys have been conducted since the 1960s in many countries and provide a hitherto untapped resource for examining trends in sedentary behaviour [9]. Previous studies have linked metabolic equivalent (MET) values to activity codes used in time use surveys allowing researchers to study the population prevalence of different activities by intensity and domain [9-12]. Research has examined population levels of physical activity and active transport using time use data [13-15], but sedentary behaviour has received less attention. Time use diaries provide a valid and reliable measurement of non-occupational sedentary behaviours [9].

In this paper, we examine trends in Australian adults’ time use by activity domain and intensity with data from the Australian Time Use Surveys in 1992, 1997 and 2006. We focus particularly on sedentary time and changes in time spent in non-occupational sedentary behaviours and more specifically on sedentary behaviours during leisure time.

## Methods

### Australian Bureau of Statistics Time Use Survey

The Australian Bureau of Statistics (ABS) conducted cross-sectional Australian Time Use Surveys in 1992, 1997 and 2006 [16-18]. The ABS randomly selected private dwellings in Australia to participate in each survey. Trained ABS interviewers collected information from one adult member of each household about all persons aged at least 20 years old in the household via computer-assisted interviews. Each person was then given time use diaries to record all their activities over two specified consecutive days. The time use diary consisted of two 24-hour activity logs with 5-minute intervals. Participants recorded their main (primary) and any accompanying (secondary) activities in their own words in their time use diaries and their responses were later coded into standard activities by trained ABS coders. Surveys were conducted throughout the calendar year by quarter. Response rates for the 1992, 1997 and 2006 Time Use Surveys were 82.9 %, 84.5 % and 82.5 %, respectively. This study had ABS approval to access the 1992, 1997 and 2006 Australian Time Use Surveys via Confidentialised Unit Record Files.

Time use diaries with over 20 activity episodes recorded per day are considered to have acceptable data quality.[19,20] The average number of episodes recorded per diary day in the three Australian Time Use Surveys for day 1 and day 2 was, respectively, 31.8 and 30.2 in 1992, 29.1 and 27.5 in 1997, and 28.9 and 27.0 in 2006, indicating good data quality in all surveys.

### Data treatment

We matched the ABS activity codes used in the 1992 and 1997 Time Use Surveys to those used in the 2006 survey so that codes would be consistent across the surveys. We then assigned to all primary activities an intensity classification based on the Compendium of Physical Activities [1] and the intensity coding system used in the American Time Use Survey.[10] The intensity classification comprised three categories defined by metabolic equivalents (MET; 1 MET = 4.184 kJ per kilogram of body weight per hour): sedentary activities (<=1.5 METs), light activities (>1.5 to <3 METs), moderate-to-vigorous activities (> = 3 METs) and occupational activities. This method for estimating non-occupational sedentary, light and moderate-to-vigorous intensity activity has been previously validated and has shown good test-retest reliability (intraclass correlations 0.74, 0.46, and 0.73, respectively) and validity when compared to accelerometers (Spearman correlations 0.57-0.59, 0.27-0.39, and 0.45-0.69, respectively) [9]. We could not code occupational activities for intensity because time at work was not broken down into specific occupational activities. We also recoded all activities into domains commonly used in physical activity research: occupational, household, leisure and transportation. Examples of sedentary household activities include clothes making, paperwork, budgeting and communications associated with domestic activities and childcare. Examples of leisure sedentary activities include attendance at movies and sports matches, games, handcraft, watching TV, and reading books. Transport modes were categorised as sedentary (car, bus, ferry, train), light (motorcycle), moderate-to-vigorous (walking, cycling) and unspecified [9]. All activities classified as moderate-to-vigorous are referred to as “moderate” activities in this paper.

Overall, the matched activity coding scheme was consistent across the three surveys. Some coding differences in 1992 meant that we could not synchronise activities in that survey with those in the 1997 and 2006 surveys in some domains. The lack of “with/for whom” information in 1992 affected the coding of social and household activities and limited comparability of leisure and household domains with subsequent surveys. Time spent in the sleep domain in 1997 and 2006 encompassed sleeping, napping and sleeplessness; however napping and sleeplessness were not coded in 1992 and so time spent in the sleep domain in 1992 was not comparable with that measured in later surveys. Hence, we could only make limited comparisons for the 1992 time use survey with those from 1997 and 2006.

New communication categories were devised to standardise and delineate communication with and without a computer. In 1992, The ABS originally coded communication as separate activities (i.e., written, in person, by computer, by phone), whereas in 1997 and 2006 they coded communication as “communication” and then coded the technology involved as a separate variable (in person, fixed phone, mobile phone, written, sms, fax, internet, personal computer). We recoded all communication involving internet or personal computers as computing and further grouped them into subcategories: computing for occupation, computing for household, computing for leisure and computing for education. All other communication modes were then coded as non-computer communication (written, in person, by phone).

### Analyses

We aggregated episode level data to the person level for analysis. Only people with two complete days of diary data were included: n = 6420 (91.0 %) in 1992; n = 6703 (92.3 %) in 1997; n = 6018 (87.2 %) in 2006. Data were weighted to the person-day to account for the probability of each person being selected by day type (week, weekend day) and then the distribution was adjusted based on population benchmarks for quarter of the year, age, sex, employment, and region of that survey year. The final weighted samples for each survey year were: n = 5851 (1992), n = 6419 (1997) and n = 5505 (2006).

We computed minutes spent over two days in activities by domain and intensity and domain-intensity combinations. Descriptive data are presented as mean and standard deviations over two complete diary days (2880 minutes). Changes in time spent in non-occupational sedentary activities across year of survey were analysed using multiple linear regressions adjusted for sex, age, education and employment status with 2006 as the reference year. The beta coefficient represents the average change per minute over two diary days in time spent in activities in 1992 or 1997 compared to 2006 (a negative coefficient indicates an increase in 2006).

## Results

Table 1 presents the weighted participant characteristics for the Australian Time Use Surveys of 1992, 1997 and 2006. While there were differences in the distributions by education and employment status across the survey years, these reflected genuine secular trends in the Australian adult population rather than artefacts of sampling [21-23].

**Table 1:** **Weighted participant characteristics by year of Australian Time Use Surveys 1992, 1997, 2006 (column %)**^**1**^

		**1992**		**1997**		**2006**	
		n	%	n	%	n	%
All		5851	100	6419	100	5505	100
Sex	Male	2906	49.7	3149	49.1	2678	48.7
	Female	2945	50.3	3269	50.9	2826	51.3
Age group	20-29	659	11.3	665	10.4	495	9.0
	30-39	1386	23.7	1417	22.1	1030	18.7
	40-49	1299	22.2	1411	22.0	1133	20.6
	50-59	927	15.8	1171	18.2	1073	19.5
	60-69	714	12.2	834	13.0	846	15.4
	70+	866	14.8	920	14.3	927	16.8
Education	High school or less	2970	50.8	3094	48.2	2666	48.4
	Diploma, certificate	2185	37.3	2169	33.8	1625	29.5
	Bachelor degree or higher	696	11.9	1156	18.0	1214	22.1
Employment status	Employed full-time	2686	45.9	2928	45.6	2491	45.3
	Employed part-time	804	13.8	979	15.2	1068	19.4
	Unemployed	441	7.5	326	5.1	133	2.4
	Not in labour force	1920	32.8	2185	34.0	1812	32.9

^1^Weighted for probability of being sampled in the Australian population by day type (week, weekend) and for population age, sex, employment status, region for survey year distributions.

Table 2 shows the minutes in primary activities by domain and intensity over two diary days for the Australian Time Use Surveys of 1992, 1997 and 2006. There was no change in time spent in the sleep or occupation domains. Total time spent in education showed some significant increases over time but the effect sizes were small (<7 minutes).

**Table 2:** **Minutes spent in primary activities by domain and intensity over two diary days, Australian Time Use Surveys 1992, 1997 and 2006**^**1**^

		**1992 (n=5851)**			**1997 (n=6419)**			**Reference year 2006 (n=5505)**	
**Domain**	**Intensity level**	**Mean (SD)**	**% time**	**B (95% CI)^2^**	**Mean (SD)**	**% time**	**B (95% CI)^2^**	**Mean (SD)**	**% time**
Sleep^3^	Total	-	-	-	1022.6 (185.0)	35.5	-0.02 (-6.4, 6.4)	1018.4 (180.8)	35.4
Occupation	Total	393.4 (470.1)	13.7	10.1 (-2.5, 22.8)	381.5 (456.8)	13.2	-2.3 (-14.6, 10.0)	391.4 (456.1)	13.6
Transport	Total	149.3 (110.7)	5.2	-0.7 (-4.8, 3.5)	148.4 (120.9)	5.2	-2.1 (-6.1, 2.0)	149.2 (111.9)	5.2
	Sedentary	119.9 (105.2)	4.2	-3.9 (-7.7, -0.10)	118.0 (107.0)	4.1	-6.7 (-10.4, -3.0)	124.3 (104.2)	4.3
	Light	0.7 (10.2)	0.0	0.10 (-0.4, 0.5)	0.7 (11.5)	0.0	0.1 (-0.3, 0.6)	0.5 (12.3)	0.0
	Moderate	14.3 (32.0)	0.5	1.4 (0.3, 2.6)	12.0 (28.3)	0.4	-0.9 (-2.0, 0.2)	12.4 (30.4)	0.4
	Unspecified mode	14.4 (39.2)	0.5	1.7 (0.2, 3.3)	17.8 (51.6)	0.6	5.4 (3.9, 6.9)	12.0 (32.0)	0.4
Leisure^3^	Total	-	-	-	580.7 (311.9)	20.2	23.2 (13.0, 33.4)	559.1 (314.1)	19.4
	Sedentary	-	-	-	509.1 (288.0)	17.7	5.1 (-4.5, 14.6)	505.7 (294.5)	17.6
	Light	-	-	-	31.1 (81.5)	1.1	12.8 (10.1, 15.4)	18.7 (65.5)	0.6
	Moderate	-	-	-	40.5 (95.4)	1.4	5.3 (1.7, 9.0)	34.8 (77.8)	1.2
Household^3^	Total	-	-	-	726.4 (361.5)	25.2	-12.5 (-23.3, -1.8)	737.8 (367.4)	25.6
	Sedentary	-	-	-	247.1 (150.8)	8.6	-3.6 (-8.5, 1.3)	251.7 (154.6)	8.7
	Light	-	-	-	350.6 (237.7)	12.2	-14.9 (-22.2, -7.7)	362.4 (238.8)	12.6
	Moderate	-	-	-	128.6 (154.0)	4.5	6.0 (0.8, 11.3)	123.7 (153.4)	4.3
Education	Total	23.0 (123.8)	0.8	-3.4 (-7.7, 1.0)	20.4 (116.1)	0.7	-6.3 (-10.5, -2.1)	24.0 (123.4)	0.8
	Sedentary	22.9 (123.5)	0.8	-3.2 (-7.5, 1.1)	20.1 (114.9)	0.7	-6.3 (-10.5, -2.2)	23.8 (122.8)	0.8
	Light	0.1 (6.3)	0.0	-0.1 (-0.4, 0.1)	0.4 (5.9)	0.0	0.1 (-0.1, 0.3)	0.3 (4.4)	0.0
	Moderate^4^	-	-	-	-	-	-	-	-
Non-occupational waking time^3^	Total^5^	-	-	-	1475.9 (439.3)	51.2	3.5 (-8.5, 15.6)	1470.2 (435.4)	51.0
	Sedentary	-	-	-	894.3 (315.9)	31.1	-11.2 (-21.5, -0.9)	905.5 (320.7)	31.4
	Light	-	-	-	382.7 (251.9)	13.3	-1.5 (-8.8, 5.8)	381.7 (247.3)	13.3
	Moderate	-	-	-	181.1 (179.6)	6.3	10.9 (4.8, 17.0)	170.9 (171.8)	5.9

^1^Weighted for probability of being sampled in the Australian population by day type (week, weekend) and for population age, sex, employment status, region for survey year distributions.

^2^ Reference year is 2006; models additionally adjusted for age, sex, education level and employment status.

^3^ Data not presented for 1992 because changes to coding in 1997 and 2006 meant that time spent in activities in sleep, leisure and household domains were not comparable.

^4^ There was no time in moderate education activities recorded.

^5^ Total non-occupational waking time includes time in transport, leisure, household and education domains.

We found no change in time spent in non-occupational activities during waking time (i.e., transport, leisure, household and education domains) from 1997 to 2006. On average, respondents spent approximately 51% of their time in non-occupational activities of which 62% was sedentary. Relative to 2006, sedentary non-occupational activity time was significantly lower in 1997 and moderate non-occupational activity time was significantly higher in 1997, both by about 11 minutes over two days.

Total time spent in transportation in both 1992 and 1997 was not significantly different to that in 2006. However time spent in sedentary modes of transport (i.e. car, bus, train) was significantly lower in 1992 (B = −3.9; 95%CI: -7.7, -0.1) and 1997 (B = −6.7; 95%CI: -10.4, -3.0) than in 2006, although again effect sizes were small. There was a small drop in time in active transport (i.e. walking and cycling) between 1992 and 2006 (B = 1.4; 95 % CI: 0.3, 2.6), while time in unspecified transport modes was significantly higher in 1992 (B = 1.7; 95%CI: 0.2, 3.3) and 1997 (B = 5.4; 95%CI: 3.9, 6.9) than in 2006.

Relative to 2006, total leisure time was significantly higher in 1997 (B = 23.2; 95%CI: 13.0, 33.4). While there was no change between 1997 and 2006 in sedentary leisure time, time in light and moderate leisure activities was significantly higher in 1997 than in 2006 (B = 12.8; 95%CI: 10.1, 15.4; and B = 5.3; 95%CI: 1.7, 9.0, respectively).

Total time spent in household activities was significantly lower in 1997 than in 2006 (B = −12.5; 95%CI: -23.3, -1.8). There was no difference in sedentary household activity time in 2006 versus 1997. Compared to 2006, time in light household activities was significantly lower in 1997 (B = −14.9; 95%CI: -22.2,-7.7) and time in moderate household activities was significantly higher in 1997 (B = 6.0; 95%CI: 0.8, 11.3).

Stratified analyses by sex (data not shown) found that there was a significant increase in total non-occupational sedentary time from 1997 to 2006 in men (1997 mean = 890.1 min/2d versus 2006 mean = 910.7 min/2d; B = −16.6; 95%CI: -31.9, -1.3), but not in women (1997 mean = 898.4 min/2d versus 2006 mean = 900.6 min/2d; B = −5.0; 95%CI: -18.8, 8.8). Compared to 2006, time in sedentary leisure activities was higher in 1997 in women (1997 mean = 498.4 min/2d versus 2006 mean = 483.2 min/2d; B = 17.0; 95%CI: 4.7, 29.2), while men showed no significant change (1997 mean = 520.3 min/2d versus 2006 mean = 529.5 min/2d; B = −5.7; 95%CI:-19.8, 8.5) Moderate leisure time decreased from 1997 to 2006 in men only (1997 mean = 51.5 min/2d versus 2006 mean = 42.5 min/2d; B = 8.9; 95%CI: 3.4, 14.3). From 1997 to 2006, women’s time spent doing household activities significantly increased (1997 mean = 871.7 min/2d versus 2006 mean = 878.1 min/2d; B = −18.0; 95%CI: -33.5, -2.6); time spent in light household activities increased (1997 mean = 469.7 min/2d versus 2006 mean = 476.8 min/2d; B = −17.4; 95%CI: -28.6, -6.3) and time in moderate household activities decreased (1997 mean = 137.0 min/2d versus 2006 mean = 130.7 min/2d; B = 6.6; 95%CI: 0.1, 13.1). Men reported significantly increased time in light household activities from 1997 to 2006 (1997 mean = 227.0 min/2d versus 2006 mean = 241.6 min/2d; B = −12.4; 95%CI: -20.1, -4.7) while there was no change in their sedentary, moderate or total household time.

Total time in all leisure-time sedentary behaviours constituted 57% and 56% of total sedentary waking hours in 1997 and 2006, respectively. Table 3 presents a breakdown of the minutes spent in leisure-time sedentary activities by primary activity. TV-viewing time did not differ between 1997 and 2006.Time spent using the computer in leisure time was almost half an hour lower in 1997 than in 2006 (B = −26.7; 95%CI: -29.5, -23.8). At the same time, there were small significant declines from 1997 to 2006 in other leisure-time sedentary behaviours: reading, relaxing and resting, listening to music, hobbies and crafts and non-computer forms of communication.

**Table 3:** **Changes in minutes spent in leisure-time sedentary behaviour (primary activities) over two diary days, Australian Time Use Surveys 1997 and 2006**^**1**^

	**1997 (n=6419)**			**Reference year 2006 (n=5505)**	
**Leisure-time sedentary behaviour**	**Mean (SD)**	**% of domain**	**B 95% CI)^2^**	**Mean (SD)**	**% of 2 domain**
Watching TV	236.9 (203.1)	46.5	3.4 (-3.4, 10.2)	231.7 (195.9)	45.8
Socialising, community interaction	71.3 (113.0)	14.0	-2.4 (-6.5, 1.8)	73.3 (115.8)	14.5
Reading	53.5 (88.7)	10.5	6.3 (3.3, 9.4)	50.5 (91.1)	10.0
Relaxing, resting	27.8 (70.8)	5.5	3.2 (0.8, 5.6)	25.9 (64.5)	5.1
Listening to music	15.1 (49.9)	3.0	6.5 (4.9, 8.1)	9.3 (38.6)	1.8
Hobbies, arts, crafts	12.1 (49.9)	2.4	2.5 (0.8, 4.3)	10.0 (44.3)	2.0
Using computer	11.8 (57.2)	2.3	-26.7 (-29.5, -23.8)	37.5 (99.5)	7.4
Communication by phone, written, in person	73.4 (93.9)	14.4	10.1 (6.9, 13.3)	62.7 (84.7)	12.4
Other	7.4 (37.4)	1.5	2.1 (1.0, 3.3)	4.8 (24.8)	1.0

^1^ Weighted for probability of being sampled in the Australian population by day type (week, weekend) and for population age, sex, employment status, region for survey year distributions.

^2^ Reference year is 2006; models additionally adjusted for age, sex, education level and employment status.

Table 4 focuses on time spent in screen-based activities and showed significantly lower total screen time in 1992 (B = −62.0; 95% CI: -69.4, -54.6) and 1997 (B = −24.2; 95%CI: -31.3, -17.0) relative to 2006. Furthermore, leisure-time computing showed a marked change over the three surveys with significantly less computer use for leisure in 1992 (B = −35.3; 95%CI: -37.7, -32.8) and in 1997 (B = −26.4; 95%CI: -28.8, -24.0) compared with 2006.

**Table 4:** **Changes in minutes spent in screen-based primary activities over two diary days, Australian Time Use Surveys 1992, 1997 and 2006**^**1**^

		**1992 (n=5851)**		**1997 (n=6419)**		**Reference year** **2006 (n=5505)**
**Screen time domain**		**Mean (SD)**	**B (95% CI)^2^**	**Mean (SD)**	**B (95% CI)^2^**	**Mean (SD)**
Total		216.7 (203.5)	-62.0 (-69.4, -54.6)	250.4 (211.6)	-24.2 (-31.3, -17.0)	271.9 (220.8)
Leisure	Watching TV, DVD, videos	214.0 (202.0)	-24.2 (-31.2, -17.2)	236.9 (203.1)	3.2 (-3.6, 10.0)	231.7 (195.9)
	Computing	2.6 (22.8)	-35.3 (-37.7, -32.8)	11.8 (57.2)	-26.4 (-28.8, -24.0)	37.5 (99.5)
Household	Computing	0.0 (1.0)	-1.7 (-2.1, -1.3)	0.8 (11.0)	-0.9 (-1.3, -0.5)	1.8 (14.9)
Education	Computing	0.1 (3.5)	-0.9 (-1.3, -0.4)	0.9 (15.3)	-0.1 (-0.6, 0.3)	1.0 (14.3)

^1^ Weighted for probability of being sampled in the Australian population by day type (week, weekend) and for population age, sex, employment status, region for survey year distributions.

^2^ Reference year is 2006; models additionally adjusted for age, sex, education level and employment status.

## Discussion

This study examined trends in Australian adults’ time use by activity domain and intensity using data from the Australian Time Use Surveys in 1992, 1997 and 2006 with particular attention given to changes in time engaged in non-occupational sedentary behaviour. To our knowledge, this is the first population study to report on temporal changes in adult non-occupational sedentary time. While previous studies have described trends in TV-viewing time, car use or walking [12,24] we present detailed prevalence data on a range of different sedentary behaviours in Australian adults over a 9- to 14-year timeframe.

Our findings suggest that non-occupational sedentary waking time has increased slightly in recent years among Australian adults. This increase was primarily due to small increases in sedentary transport and education time, while sedentary household and leisure time remained stable. We also found that the distribution of different leisure-time sedentary activities changed, while total sedentary leisure time remained stable. For example, leisure-time computer use showed a significant increase from 1997 to 2006, but other leisure-time sedentary behaviours (e.g., reading, listening to music, hobbies and crafts) showed small reductions concurrently. Total screen time increased over the 14 years between 1992 and 2006, again mostly due to growth in computer use.

Many of the studies on the adverse effects of sedentary time on health are based on watching television [25-28]. A recent meta-analysis showed that prolonged TV-viewing is associated with higher risk of type 2 diabetes, fatal and non-fatal cardiovascular disease and all-cause mortality.[29] In the present study, there was only a modest increase in watching television between 1992 and 2006, but the increase in total screen time was more substantial and might be of public health significance. Our results also showed that 90% of leisure time in 2006 was spent sedentary and over half (53%) of that consisted of screen time. It is likely that these upward trends in screen time will continue given the increasing reliance of modern life on computer technology. Therefore, studies of the associations between these types of sedentary behaviours and health outcomes are warranted, especially as much of the focus to date has been on TV-viewing time. Furthermore, these data suggest that reducing TV-viewing and other screen time may be good targets for public health interventions. Advances in technology may complicate future research with less distinction between types of sedentary behaviours as they become increasingly screen-based with the development of smart phones and tablet devices.

We found that time using sedentary modes of transport rose slightly between 1992 and 2006, while time in moderate intensity transport modes showed a small concurrent decline. There were also small but significant increases in time spent in unspecified modes of transport for which activity intensity could not be determined and makes the drawing of conclusions more difficult. Findings from Australian and American studies suggest that the prevalence of car use is high and has increased, while active transport, with relatively low prevalence, has changed little [24,30,31]. Built environment and transport policies and active transport promotion can influence active travel [32,33]. It is possible that use of active transport modes may increase in the future given growing interest and calls to action in Australia about implementing new transport and built environment infrastructure and policies to encourage more active transport [34-37]. Further monitoring through analyses similar to those presented here could track such changes.

We found that total time in household activities increased in women but not in men from 1997 to 2006. Women’s time in sedentary household tasks did not change over this period, but time doing light household activities increased while that for moderate household activities decreased. It is possible that the decline in moderate household activities is due to increased consumption of labour-saving goods and services over this period [38].

It is not clear whether the changes in sedentary time found in this study could affect health outcomes as research is yet to establish thresholds for sitting time which compromise health [39]. There are currently no official adult guidelines for sedentary behaviour, although the Australian National Heart Foundation recommends limiting screen-based activities to less than two hours per day [40].

### Strengths and limitations

The key strength of this study is the use of data from three cross-sectional population-based time use surveys. The response rates from each survey were relatively high (all >80%) and data quality was also high (average number of episodes recorded per diary day ranged from 27 to 31). [19,20] A further advantage is that time use surveys are not health-focused. Participants report all of their time use over the diary measurement period and they are not explicitly asked to focus on their physical activity or sedentary behaviour. So, potential social desirability related to physical activity and sedentary behaviour when completing time use surveys is likely to be low,[13] resulting in less reporting bias than in other self-report measures of physical activity and sedentary behaviour, which was illustrated in a previous comparison of time use diaries against accelerometers [9].

This study was limited by the lack of detail about occupational activities reported in the Time Use Surveys. We found that there were no changes in time spent in the occupational domain from 1992 to 2006, but could not determine the intensity level of occupational activities and the possible changes in sedentary, light and moderate occupational activities over the survey years. It is possible that time spent in sedentary and light occupational activities have risen while that for moderate occupational activities declined, in light of population trends in the US and England [41-43]. Thus, total sedentary time during waking hours in Australian adults would probably be higher if potential occupational sedentary time could have been factored in.

Changes in activity coding after 1992 limited the analyses of trends in the sleep, household and leisure domains to comparing changes between 1997 and 2006 Time Use Surveys only. We were not able to compare time spent in leisure and household activities in 1992 with that in 1997 and 2006 due to the lack of “with/for whom” information in 1992 which affected the coding of social and household activities. Nonetheless, we were able to standardise codes for computer use in all three surveys which allowed for the analysis of trends in screen time across a 14-year period. Other studies have reported issues with changes in coding systems in time use surveys over several cycles and researchers have similarly harmonised data to examine changes in time spent in certain behaviours over time [11,12].

## Conclusions

Australian adults’ time engaged in non-occupational sedentary activities has increased slightly from 1997 to 2006. This appears to be the result of small increases in sedentary transport and education time while sedentary household and leisure time were stable over this time period. However, 90% of leisure time is spent sedentary and the composition of sedentary leisure time changed between 1992 and 2006, with the proportion of time devoted to TV-watching and leisure-time computer use increasing over 14 years. As the literature has focused on the detrimental effects of TV-viewing and health, these increases in screen time have serious public health implications. With almost all leisure-time spent sedentary, leisure-time seems a suitable target for public health interventions aimed at reducing sedentary behaviour as the potential gains would be substantial. Future research should focus on the detrimental effects of different types of sedentary behaviour in order to determine if certain sedentary behaviours such as TV-viewing are more harmful than others.

This study makes a unique contribution in that we report trends in a range of different sedentary behaviours by domain in a population-representative sample of Australian adults. It also demonstrates the utility of time use surveys for studying trends in non-occupational sedentary behaviours when time trend data on population prevalence of sedentary behaviours are limited.

## Competing interests

The authors declare that they have no competing interests.

## Authors' contributions

JYC and HPVDP conceptualised the study and research questions. JYC obtained permission for data access, analysed and interpreted the data, and drafted the manuscript. HPVDP, DM and AG contributed to data analysis and provided statistical advice. HPVDP, CR, AEB, DM, AG contributed to interpreting the data. All authors critically revised the manuscript for intellectual content, read and approved the final manuscript.
